# Immune dysregulation from a novel CTLA-4 haploinsufficiency variant

**DOI:** 10.70962/jhi.20250112

**Published:** 2025-12-26

**Authors:** Nina N. Brodsky, Alan Kennedy, Daniel Glaser, Lauren Jeffries, Weizhen Ji, Eesha Natarajan, Junghee J. Shin, David M. Sansom, Carrie L. Lucas, Saquib A. Lakhani

**Affiliations:** 1 Department of Pediatrics, Pediatric Genomics Discovery Program, Yale University School of Medicine, New Haven, CT, USA; 2 Department of Immunobiology, Yale University School of Medicine, New Haven, CT, USA; 3 UCL Institute of Immunity and Transplantation, London, UK; 4 Department of Internal Medicine, Rheumatology, Allergy and Immunology, Yale University School of Medicine, New Haven, CT, USA; 5 Department of Pediatrics, Cedars Sinai Guerin Children’s, Los Angeles, CA, USA

## Abstract

Cytotoxic T lymphocyte antigen 4 (CTLA-4) is a key immune checkpoint receptor that regulates T cell activation through ligand competition and transendocytosis. Heterozygous loss-of-function variants in CTLA4 result in CTLA-4 haploinsufficiency with autoimmune infiltration (CHAI), characterized by immune dysregulation and autoimmunity. We report a multigenerational family carrying a novel heterozygous CTLA4 variant, c.654T>A (p.Tyr218*), which truncates the cytoplasmic tail. Affected individuals presented with recurrent infections and autoimmune manifestations. Patient T cells showed reduced CTLA-4 expression at baseline and after stimulation, suggesting impaired stability. Jurkat cells expressing CTLA-4 Y218* exhibited enhanced degradation, partially rescued by lysosomal inhibition, and reduced transendocytosis of CD80. Together, these findings suggest that the CTLA-4 p.Tyr218* variant compromises protein stability and ligand uptake, contributing to CTLA-4 haploinsufficiency and immune dysregulation. This work broadens the spectrum of CTLA4 variants and underscores the importance of the C-terminal cytoplasmic domain in CTLA-4 function and immune regulation.

## Teaser

We describe a novel CTLA-4 p.Tyr218* variant associated with immune dysregulation across four generations. The mutation truncates the cytoplasmic tail, reducing CTLA-4 stability and transendocytosis, consistent with haploinsufficiency and autoimmunity. These findings suggest an important role for the C-terminal domain of CTLA-4 in maintaining immune homeostasis.

## Graphical Abstract

**Figure:**
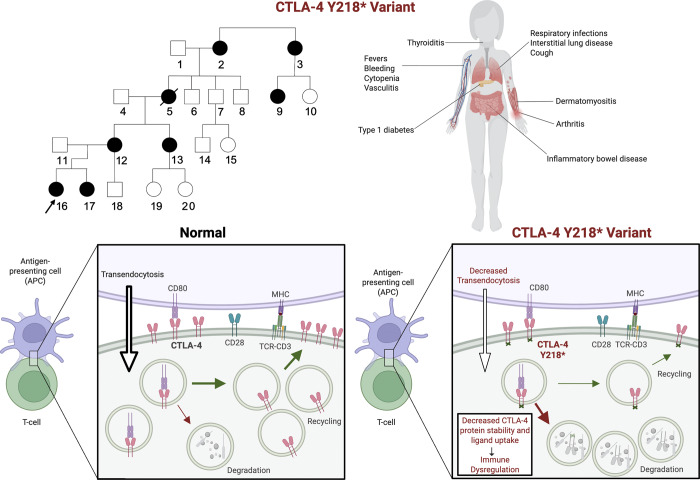

## Introduction

Cytotoxic T lymphocyte antigen 4 (CTLA-4) is an immune checkpoint receptor that plays a central role in regulating T cell responses (1, 2). Primarily expressed in activated T cells and regulatory T (T_reg_) cells, the surface expression of CTLA-4 is tightly controlled. CTLA-4 inhibits T cell activation by binding with high affinity to the co-stimulatory B7 ligands, CD80 and CD86, expressed on antigen-presenting cells (APCs) and thereby prevents their binding to the T cell co-stimulatory receptor CD28. In addition to competitive inhibition, CTLA-4 mediates transendocytosis (TE), a process by which it removes CD80/CD86 molecules from the surface of APCs, internalizing them into the T cell to further diminish co-stimulatory signals. These mechanisms maintain immune homeostasis by preventing excessive T cell activation.

Heterozygous loss-of-function *CTLA4* variants cause CTLA-4 haploinsufficiency with autoimmune infiltration (CHAI), a disorder manifesting with a combination of autoimmunity and immunodeficiency (1, 2). In CHAI, failure of CTLA-4 to mediate its inhibitory effect results in T cells that are hyper-stimulated, leading to lymphoproliferation and lymphocytic tissue infiltration. Common manifestations include recurrent respiratory infections, inflammatory bowel disease, lymphocytic infiltrates, autoimmune cytopenias, and type 1 diabetes (1, 2, 3). Elucidating the mechanisms by which CHAI variants affect CTLA-4 function, stability, and TE may yield insights into the pathogenesis of immune-related disorders and inform the development of personalized therapeutic strategies.

## Results

Here, we report a patient who presented with recurrent infections and inflammation at the age of 2 years. She experienced frequent respiratory viral infections requiring hospitalization during infancy, along with an episode of noninfectious transient coagulopathy. At 1 year of age, she developed recurrent episodes of fever, petechial rash, diarrhea, and abdominal bloating. Growth and development during this period were normal. She continued to experience respiratory infections, prolonged bleeding with transient coagulopathy without thrombocytopenia, and intermittent bloody diarrhea throughout her second year of life. At 18 mo of age, clinical exome sequencing reported no diagnostic variants. Mitochondrial genome analysis performed due to intermittently elevated lactate (as high as 4.6 mmol/L; reference 0.5–2 mmol/L) was normal. At ∼2 years of age, flow cytometric analysis demonstrated normal lymphocyte subsets, including CD3^+^ T cells (3,475 cells/μl; reference range 2,100–6,200 cells/μl), CD19^+^ B cells (744 cells/μl; reference 720–2,600 cells/μl), and class-switched memory B cells (CD19^+^CD27^+^IgD^−^IgM^−^, 9.8%; reference not established). Immunoglobulin concentrations were within normal limits: IgG 650 mg/dl (reference 330–1,090 mg/dl), IgM 49 mg/dl (reference 19–146 mg/dl), IgA 28 mg/dl (reference 20–100 mg/dl), and IgD 15 mg/dl (reference <179 mg/dl). Vaccine antibody titers were normal or indeterminate, including *Streptococcus pneumoniae* serotypes (2.3–25.6 µg/ml; protective ≥1.3 µg/ml), *Haemophilus influenzae* type b (0.46 µg/ml; reference 0.15–0.99 µg/ml = indeterminate; ≥1 µg/ml = protective), and tetanus antitoxoid (0.11 IU/ml; protective >0.10 IU/ml). Of note, soluble IL-2 receptor levels were elevated (4,865 pg/ml; reference 532–1,891 pg/ml), consistent with activation of adaptive immunity. She was started on prednisone and canakinumab (an IL-1β–blocking antibody; 6 mg/kg every 4 wk) with partial improvement in her fever episodes. A gastrostomy tube was placed at 26 mo due to recurrent unexplained hypoglycemia and poor growth. Additionally, she developed a chronic cough, partially responsive to inhaled corticosteroids, and recurrent urinary tract infections. Urologic testing revealed bilateral vesicoureteral reflux, which was corrected surgically. At 28 months, research analysis of clinical exome data identified a novel (absent in gnomAD v.4.1.0) heterozygous variant in *CTLA4*, MANE Select transcript NM_005214.5: c.654T>A, p.(Tyr218*), located in exon 4 in the C-terminal cytoplasmic tail. Review of the prior clinical exome results revealed that the diagnostic lab interpreted this *CTLA4* variant as being of uncertain significance and not clinically reportable at the time. The lab also noted two variants of uncertain significance, p.(Arg408Gln) and p.(Pro369Ser), in *MEFV*, which encodes for pyrin. These variants were in cis and both paternally inherited, making recessive disease unlikely. Additionally, both were present at >5% in subpopulations from the gnomAD database and classified as polymorphisms unlikely to cause autosomal-dominant disease.

Given the presence of the *CTLA4* variant, subcutaneous abatacept was initiated at 30 mo. Abatacept is a fusion protein of the extracellular domain of CTLA-4 combined with the Fc region of IgG1 that competitively binds to and reduces signaling through CD80 and CD86, thus inhibiting co-stimulation. A dose of 125 mg weekly had a good clinical response, including resolution of the cough. Supplemental intravenous immunoglobulin therapy started at 33 mo reduced the frequency of viral infections. Canakinumab was stopped, and oral sirolimus (an mTOR inhibitor; 2 mg daily) was added at 45 mo, after which glycemic levels stabilized and energy levels improved without clear mechanistic explanation. Episodes of mouth ulcers returned, prompting transition to intravenous abatacept (40 mg/kg every 4 wk) with good response. She is currently pending allogenic hematopoietic stem cell transplant.

**Figure 1.**
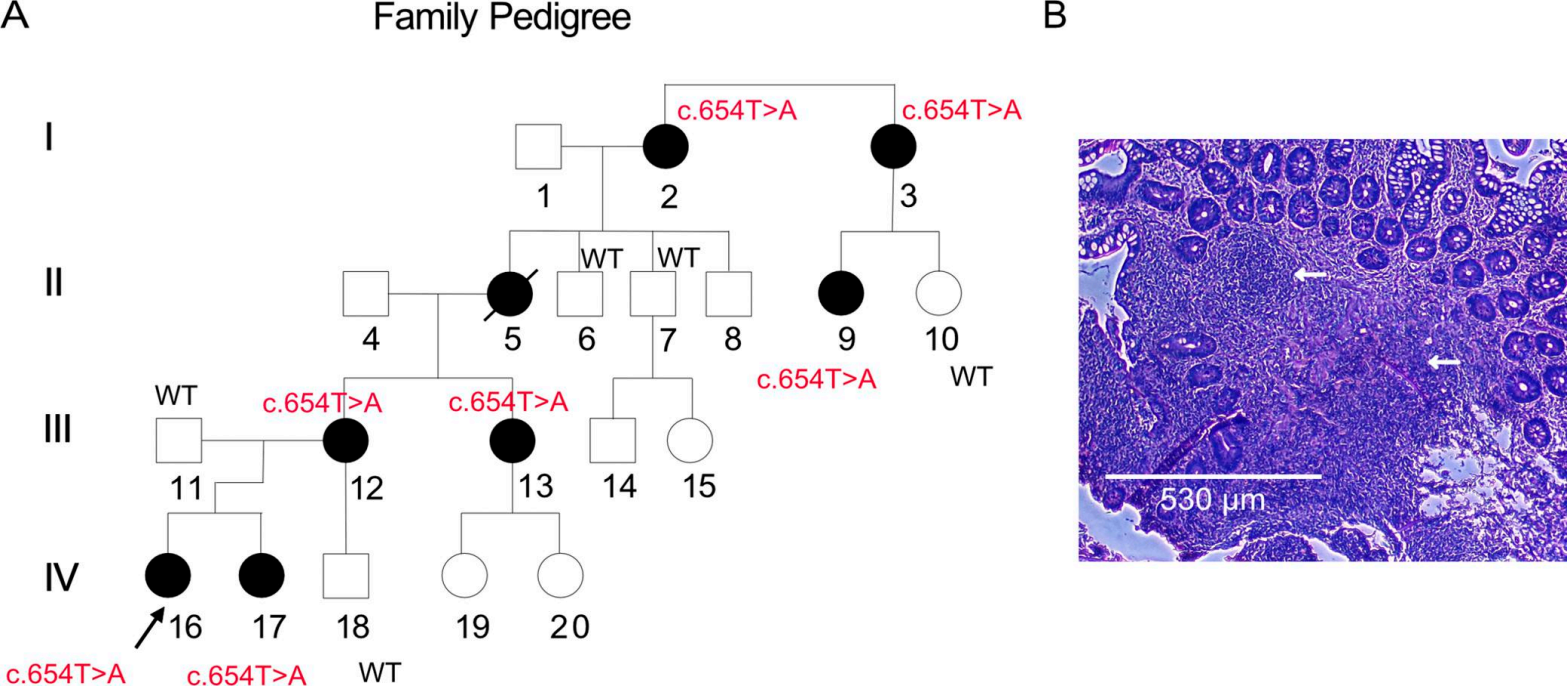
CTLA-4 Y218* mutation in four generations of a family. (A) Pedigree of the affected family, with proband identified by the arrow (patient 16); filled symbols signify clinically affected family members, with the heterozygous *CTLA4* c.654T>A, p.(Tyr218*) variant noted in individuals who harbor it. WT denotes family members sequenced and found to have no mutation. Squares represent males, and circles represent females; a diagonal line (as in family member 5) indicates a deceased individual. **(B)** Biopsy of the terminal ileum from P12 showing diffuse lymphocytic infiltrates (arrows).

Sanger sequencing of additional family members revealed the c.654T>A p.(Tyr218*) variant in seven females across four generations, all of whom suffer from autoimmunity and/or recurrent infections (Fig. 1 A). The proband’s mother (P12) has a history of kidney infections, gestational and type 1 diabetes, arthritis, Sjogren’s syndrome, Hashimoto’s thyroiditis, gastrointestinal disease (Fig. 1 B), and prolonged bleeding (e.g., heavy menstrual periods, epistaxis, and bleeding with appendectomy). Her sister (P17) experiences recurrent infections, febrile episodes, joint hypermobility, and positive autoantibodies associated with type 1 diabetes. Her maternal aunt (P13) has hypermobile Ehlers-Danlos syndrome, dermatomyositis, arthritis, and interstitial lung disease. Her maternal grandmother (P5) had cytopenia, hypocomplementemic urticarial vasculitis, required a hysterectomy due to heavy menstrual bleeding, and died at age 50 from presumed cardiac arrest. Her maternal great-grandmother (P2) suffers from pancytopenia, ulcerative colitis, Hashimoto’s thyroiditis, dermatomyositis, recurrent infections, and pernicious anemia. The maternal great-grandmother’s sister (P3) suffers from rheumatoid arthritis, dermatomyositis, and ulcerative colitis, and the maternal great-grandmother’s niece (P9) suffers from rheumatoid arthritis, alopecia, inflammatory bowel disease, Hashimoto’s thyroiditis, and severe anemia.

**Figure 2.**
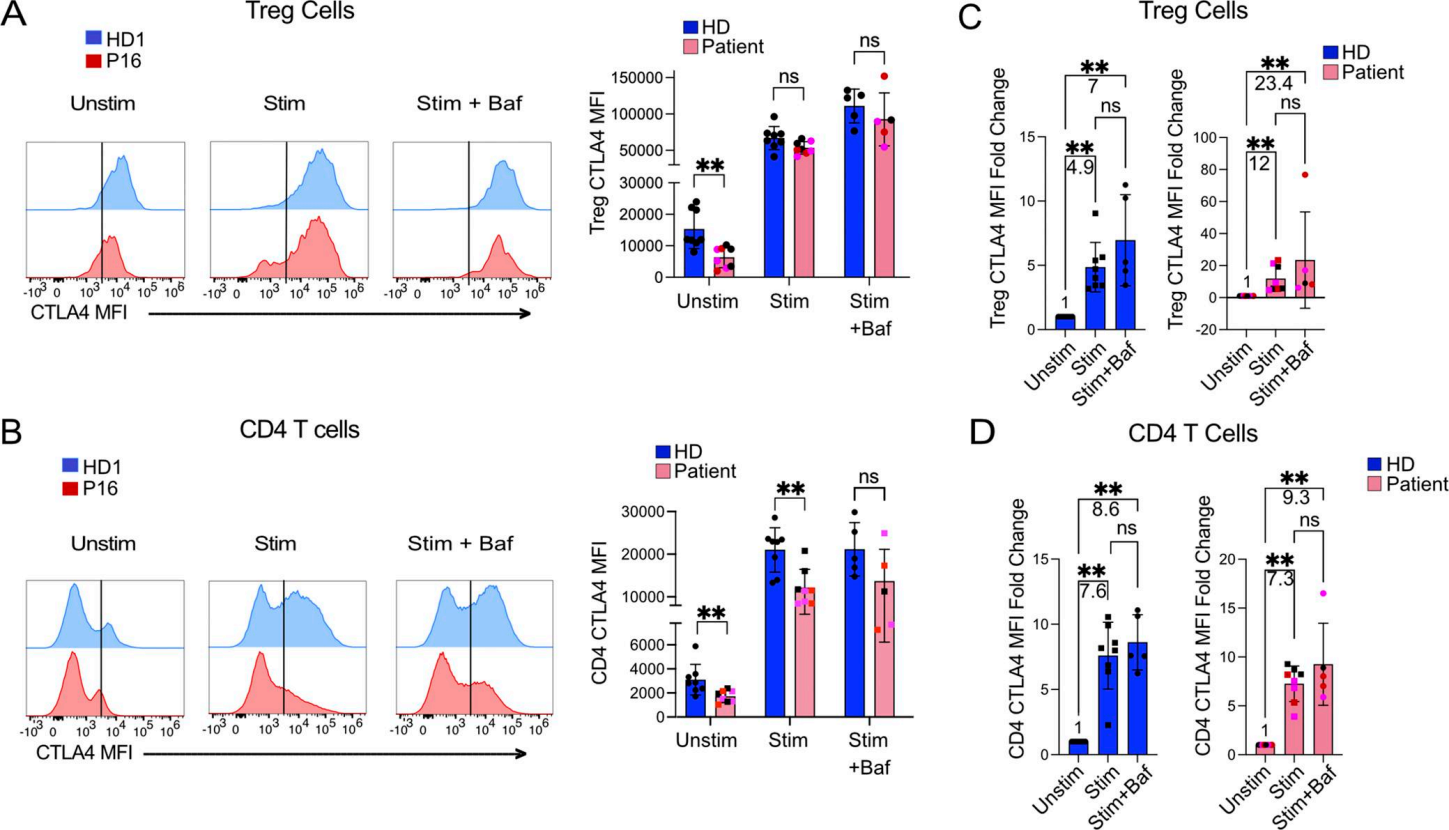
CTLA-4 Y218* mutation leads to reduced protein levels in primary patient cells. (A) Representative histograms of CTLA-4 mean fluorescence intensity (MFI) levels in T_reg_ cells in one healthy donor and P16 with p.(Tyr218*) variant after 24 h of incubation in IL-2 media alone (Unstim), with stimulation (5 μg/ml plated anti-CD3 and 1 μg/ml soluble anti-CD28; Stim) or stimulation and Baf treatment (20 ng/ml; Stim + Baf); quantified MFI on the right using multiple Mann–Whitney tests (*N* = 3 independent repeats). **(B)** Representative histograms of CTLA-4 MFI in CD4 T cells of one healthy donor and P16 after 24 h of incubation in IL-2 media alone (Unstim), with stimulation (5 μg/ml plated anti-CD3 and 1 μg/ml soluble anti-CD28; Stim) or stimulation and Baf treatment (20 ng/ml; Stim + Baf); quantified MFI on the right using multiple Mann–Whitney tests (*N* = 3 independent repeats). **(C)** Quantified fold-change data from A, normalized to the unstimulated condition. Statistical significance calculated using Kruskal–Wallis test. **(D)** Quantified fold-change data from B, normalized to the unstimulated condition. Statistical significance calculated using Kruskal–Wallis test. **(A–D)***N* = 3 independent experiments with eight healthy donor (HD) samples (eight individuals) and eight patient samples (five patients: P16, P17, P12, P13, and P2). P16 and P12 contributed longitudinal samples at multiple time points (red = P16; pink = P12), with only one sample per individual used in any single experiment. Each black dot represents a distinct healthy donor or patient sample. **P < 0.001. number represents fold change. ns: not significant.

The *CTLA4* p.(Tyr218*) variant terminates the protein (NP_005205.2) five amino acids early and occurs at a tyrosine site that potentially mediates interactions with protein phosphatase 2A (PP2A) (4). Without PP2A binding, AKT activity—and consequently T cell activation capacity—would be expected to increase. Notably, Tyr218 has also been reported to be critical for CD86 TE in mouse B cells (5). To explore the molecular impact of the p.(Tyr218*) variant, we obtained whole blood from five affected females (P2, P12, P13, P16, and P17) and eight healthy female donors. Affected patients exhibited significantly decreased CTLA-4 levels in both total CD4^+^ T cells and T_reg_ cells (CD4^+^CD25^+^FOXP3^+^) at baseline (Fig. 2, A and B). After stimulation of peripheral blood mononuclear cells (PBMCs) with plate-bound anti-CD3 and soluble anti-CD28, with or without bafilomycin A1 (Baf; an inhibitor of lysosomal acidification and autophagosome-lysosome fusion), CTLA-4 levels were increased in both patient and control T_reg_ and CD4 T cells. Addition of bafilomycin inhibits degradation of internalized CTLA-4, allowing higher levels of the protein to recirculate back to the cell surface. CTLA-4 levels were significantly reduced among total CD4^+^ T cells from patients under both unstimulated and stimulated conditions (Fig. 2 B). Notably, T_reg_ cells from patients demonstrated a higher fold change in CTLA-4 expression after stimulation, with or without Baf treatment (Fig. 2 C). This may represent a compensatory mechanism to increase production of CTLA-4 in T_reg_ cells to counteract the lower baseline levels and stability of the protein in patients. In contrast, the fold change of CTLA-4 expression in total CD4^+^ T cells after stimulation, with or without Baf treatment, was similar between patients and healthy controls (Fig. 2 D).

**Figure 3.**
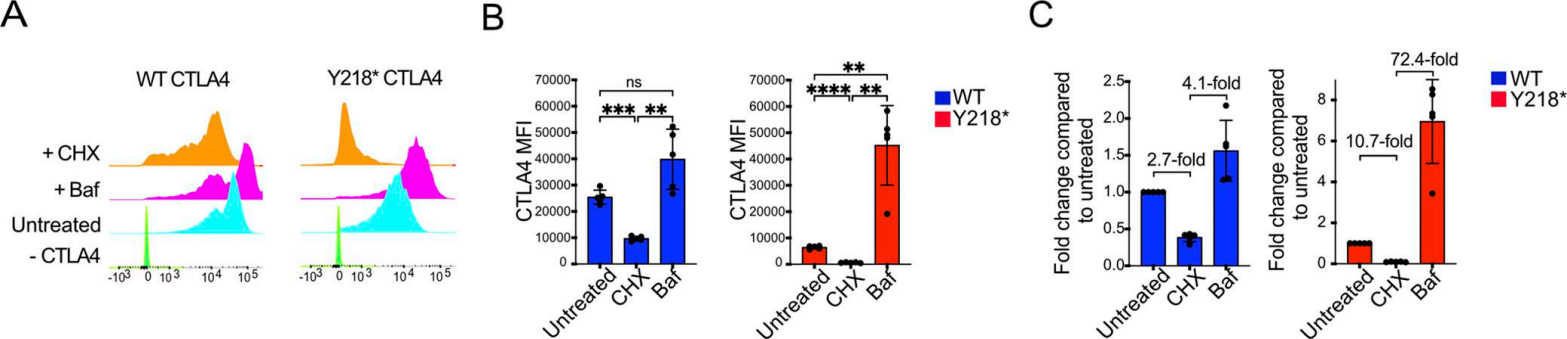
CTLA-4 Y218* mutation leads to protein instability. (A–C) (A) Representative histogram showing MFI of CTLA-4 levels in Jurkat cells with WT or Y218* CTLA-4 treated with CHX to block protein translation, Baf to block lysosome acidification, or untreated. Jurkat cells lacking CTLA-4 (−CTLA4) shown as negative control. MFI quantified in B, and fold change quantified in C; the y-axis in C shows fold change relative to the untreated condition. The mutant panel uses a different scale due to higher fold-change values compared to WT. Fold-change values in the statistical comparison bar represent relative differences between the indicated conditions; repeated measures one-way ANOVA test. *N* = 5 independent repeats. **P < 0.001, ***P < 0.001, and ****P < 0.0001; ns: not significant; number represents fold change.

**Figure 4.**
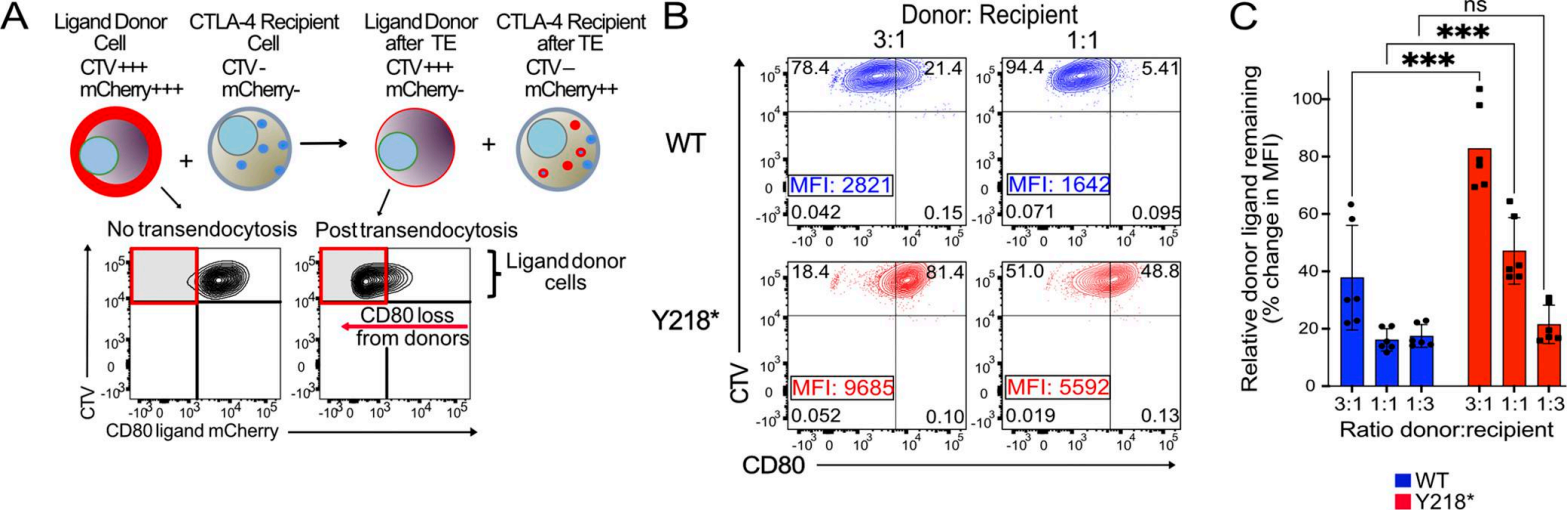
CTLA-4 Y218* mutation leads to dysfunctional TE. (A–C) (A) Experimental design and (B) representative dot plot of CD80 donor levels following TE by Jurkat recipient cells with WT (blue blots) or Y218* CTLA-4 mutation (red blots), with relative ligand loss quantified in C using multiple unpaired *T* test. *N* = 6 independent repeats for each condition. ***P < 0.001; ns: not significant.

To understand the role of the p.(Tyr218*) variant in CTLA-4 protein stability and TE, we expressed wild-type (WT) or mutant CTLA-4 (Y218*) in Jurkat cells that do not express CTLA-4 at baseline. The Y218* cell line exhibited a significant deficiency in CTLA-4 levels in the untreated state, which was exacerbated by cycloheximide (CHX, a translation inhibitor used to impair new CTLA-4 transcripts from translation into protein) treatment and rescued by Baf treatment. We observed markedly higher fold-change increase in CTLA-4 levels between CHX- and Baf-treated mutant cells (72.4-fold increase) compared to corresponding WT CTLA-4 Jurkat cells (4.1-fold increase) (Fig. 3, A–C). These data suggest that the mutant CTLA-4 is more prone to degradation and dependent on translation of new protein than the WT counterpart, although further studies are warranted to calculate rates of decay for the Y218* mutant. Upon testing TE, Y218* CTLA-4 recipient Jurkat cells showed reduced ability to remove CD80 from donor cells. Increasing recipient cells, represented as the donor-to-recipient cell ratio, was able to rescue this phenotype (Fig. 4, A–C).

## Discussion

In sum, these findings provide evidence that the novel p.(Tyr218*) CTLA-4 variant causes haploinsufficiency and immune dysregulation. Most known disease-causing variants in CTLA-4 are found in or near the extracellular and transmembrane domains, which are critical for surface expression and ligand binding (1, 2). Interestingly, there are reports of C-terminal CTLA-4 variants associated with cancer (6) but not CHAI. Notably, a phenotypically similar syndrome referred to as LATAIE (LRBA [lipopolysaccharide responsive beige-like anchor] deficiency with autoantibodies, T_reg_ cell defects, autoimmune infiltration, and enteropathy) shares many clinical features with CHAI and may more closely align with the proband’s clinical manifestations (7). The LRBA protein binds to CTLA‐4’s C-terminal cytoplasmic tail and prevents its lysosomal trafficking and degradation (7, 8).

The location of the novel p.(Tyr218*) variant is consistent with the importance of the C-terminal cytoplasmic tail in conferring CTLA-4 protein recycling, stability, and function, including TE. To further contextualize these findings, the differing effects of bafilomycin between Jurkat cells and primary patient T cells likely reflect both biological and technical factors. Jurkat cells, as a transformed line maintained under uniform culture conditions, may exhibit a more pronounced and readily detectable accumulation of CTLA-4 following lysosomal inhibition. In contrast, primary PMBCs display greater biological variability and heterogeneous activation states, and our study may not have been powered to detect modest changes in CTLA-4 levels in primary T cells. External factors such as ex vivo stimulation conditions/kinetics, cytokine milieu, or receptor turnover dynamics may also differentially influence CTLA-4 trafficking in primary versus Jurkat cell experiments.

Although the striking pedigree of this family, together with the features and molecular data presented here, support the deleterious effects of the p.(Tyr218*) variant, further investigation is needed to elucidate phenotype–genotype relationships and to refine classification of CHAI features in relation to variant location. Furthermore, more work is needed to understand broader immune system, hemostasis, and metabolic sequelae attributable to this variant, as well as drivers of heterogenous phenotypes (incomplete penetrance) of affected individuals, including modifying variants in other genes and the effects of age, microbiome, and the environment.

## Materials and methods

### Human subjects research

This study was conducted in accordance with the Declaration of Helsinki and was approved by the Yale Human Research Protection Program Institutional Review Boards (IRB). Informed consent was obtained from all participants prior to sample collection. Peripheral blood was collected from consented patients (females ages 4–96; mean 34) and healthy donors (females ages 20–64; mean 35) at Yale and collected in Acid Citrate Dextrose (ACD) blood collection tubes. All human subjects in this study provided informed consent to use their samples for research and to publish de-identified genetic sequencing data. All relevant ethical regulations for work with human participants were followed. Blood samples were processed within 24 h of collection.

### Whole-exome and Sanger sequencing

Genomic DNA was prepared from peripheral blood using standard procedures. The whole exome was captured using xGen target kit from IDT, and 101 base paired-end sequencing on the Illumina platform (NovaSeq 6000) was performed. We acquired the clinical sequencing data of the proband, which were clinically tested under Clinical Laboratory Improvement Amendments (CLIA) protocol at Yale DNA diagnostic lab, and Pediatric Genomic Discovery Program (PGDP) sequenced the parents’ DNA samples under a research protocol at the Yale Center for Genome Analysis (YCGA), after the family was consented for research under Yale IRB approval. The sequence reads were converted to FASTQ format and were aligned to the reference human genome (hg19). Genome Analysis ToolKit (GATK) best practices were applied to identify genetic variants, and variants were annotated by ANNOVAR. Data are accessed through the Yale High-Performance Computing system for analysis with our established pipelines, which are supported by YCGA. The proband sample was sequenced to a mean of 250× coverage, and the parents’ samples were sequenced to a mean of 50× coverage, with at least 20 independent reads in 98.6% (proband) and 95% (parents) of targeted bases. Whole-exome sequencing data for P18 and his mother (P12; duo) was obtained using a NimbleGen VCRome capture kit and sequenced on an Illumina HiSeq 2500 platform.

Exonic or splice-site rare variants (Minor Allele Frequency [MAF] ≤ 0.01) that exhibited high-quality sequence reads were analyzed. The rare non-synonymous variants were prioritized if they had higher Combined Annotation Dependent Depletion (CADD) scores or were predicted to be deleterious on the gene or gene product by multiple in silico predictions. De novo, X-linked, dominant, or recessive inheritance modes were in consideration for family-based genetic analysis. A panel of ∼500 self-assembled immune-related genes was analyzed as well. The *CTLA4* c.654T>A variant from the proband (P16) and the mother (P12) were called as heterozygous with 48–49% variation from a total of >760 reads in the proband sample and >48 reads in the mother’s sample. The genotypes of family members 2, 3, 6, 7, 9, 10, 12, 13, 17, and 18 (Fig. 1 A) were examined by Sanger sequencing.

### Blood processing

PBMCs were isolated using density gradient centrifugation. Blood samples were diluted 1:1 with phosphate-buffered saline (PBS) and carefully layered onto lymphoprep medium (STEMCELL) in a 50-ml conical tube. Following centrifugation for 20 min at 20°C at 824 Relative Centrifugal Force/g-force (RCF/G) with no deceleration, the PBMC layer was collected and washed twice with complete RPMI media.

### Cell culture

PBMCs (2 million cells/2 ml) were cultured in 24-well plates coated with anti-CD3 antibodies (clone: OKT3, catalog no. 317326; BioLegend) at a concentration of 5 μg/ml (coated overnight at 4°C and washed twice with PBS prior to cell plating). Soluble anti-CD28 (catalog no. 302943; BioLegend, 1 μg/ml) was added to the cultures to provide a second signal for T cell activation and proliferation. Cells were activated and maintained in complete RPMI-1640 media supplemented with 10% FBS and 1% penicillin-streptomycin and 200 U/ml IL-2 (Prometheus).

### Viability staining

Cells were stained with fixable viability stain (FVS) 780 (1:2,000 in PBS; BD Biosciences) and Human TruStain Fc block (1:50; BioLegend) at a concentration of 2 million cells in 100 μl antibody mix for 10 min at room temperature prior to flow cytometry staining to detect viable cells and complete Fc block.

### Flow cytometry

For flow cytometric analysis, PBMCs were stained with a panel of fluorochrome-conjugated monoclonal antibodies specific for surface markers in FACS buffer (PBS with 2 mM EDTA and 5% BSA, pH 8.5). The antibodies were used in a 1:100 dilution and included CD8a-BV510 (catalog no. 3011048; BioLegend), CD4-PE/Cy7 (catalog no. 300512; BioLegend), and CD25-PB (catalog no. 356129; BioLegend).

For intracellular staining, the cells were first stained with FVS 780, Fc block, and surface markers and then fixed/permeabilized with Foxp3 Fixation/Permeabilization buffer (Thermo Fisher Scientific) as per the manufacturer’s protocol. Cells were stained with 100 µl of conjugated antibody mixture in 1X permeabilization buffer for detection of intracellular proteins and incubated for 60 min at room temperature in the dark. Cells were washed with permeabilization buffer and resuspended in FACS buffer for flow cytometric analysis using Cytek Northern Lights flow cytometer and MACSQuant. Antibodies used at 1:10 dilution included: FOXP3-PE (catalog no. 560046; BD) and CTLA-4/CD152-APC (catalog no. 555855; BD).

### Cell line engineering

Jurkat cells were transduced using virus produced by co-transfection of Phoenix-A cells with either CTLA-4 WT or CTLA-4 Y218* sequences cloned into the MP71 retroviral vector and pVSV-G. Retroviral supernatants were harvested 24 h after transfection. For transduction, non-tissue culture-treated 24-well plates were coated with 30 mg/ml RetroNectin (Takara) overnight at 4°C. 5 × 10^5^ cells were added to 1 ml of retroviral supernatants in the RetroNectin pre-coated wells and centrifuged at 2,000 rpm at 32°C for 90 min. 24 h after infection, media was changed to fresh RPMI complete media. 3 days after transduction, cells were screened by staining for transduced protein expression and analyzed by flow cytometry.

### Protein stability assays

Jurkat cells expressing CTLA-4 WT or Y218* were treated with 20 μg/ml CHX or 50 nM Baf in media overnight. Following treatment, cells were fixed with 4% PFA in PBS. The cells were then permeabilized with 0.1% saponin in PBS and stained with anti-CTLA-4 PE (BD Biosciences, clone BNI3) in PBS with 0.1% saponin for 30 min. The cells were then washed and analyzed.

### TE assays

For TE assays by flow cytometry, CD80mCherry-expressing donor cells were labeled with CellTrace Violet labeling kit (Thermo Fischer Scientific) according to the manufacturer’s instructions. Recipient Jurkat cells expressed either CTLA-4 WT, Y218* CTLA-4 mutation, or no CTLA-4.

Donor and recipient cells or CTLA-4–negative control cells were plated in round-bottom 96-well plates at 37°C at the ratio of donor:recipient cells indicated in the figure legend for 21 h. Wells were supplemented with 10 ng/ml of Staphylococcal Enterotoxin E (Biomatik) to enhance cell–cell contacts. For analysis, singlets were gated, and % mCherry level reduction in donors (CTV^+^) in the presence of CTLA-4 relative to CTLA-4–negative conditions was assessed by FACS.

### Statistical methods

Multiple Mann–Whitney tests were used to determine significant mean differences between patient and healthy donor CTLA-4 levels in primary T cells. Kruskal–Wallis tests were used to compare CTLA-4 levels in differently treated patient or healthy donor primary cells. Repeated measures one-way ANOVA test was used to compare CTLA-4 levels in differently treated Jurkat cells (either with WT or Y218* mutant CTLA-4), and multiple unpaired *T* test was used to determine significant differences in the amount of donor ligand present in cells after treatment with either mutant or WT CTLA-4 Jurkat cells compared to donor ligand levels in the absence of CTLA-4, which are calculated as 100% ligand remaining. GraphPad Prism was used to perform the analysis.

## Data Availability

Variant data have been submitted to ClinVar, accession number SCV006550823.
